# 8-year trends in physical activity, nutrition, TV viewing time, smoking, alcohol and BMI: A comparison of younger and older Queensland adults

**DOI:** 10.1371/journal.pone.0172510

**Published:** 2017-03-01

**Authors:** Stephanie J. Alley, Mitch J. Duncan, Stephanie Schoeppe, Amanda L. Rebar, Corneel Vandelanotte

**Affiliations:** 1 Physical Activity Research Group, School of Medical, Health and Applied Sciences, Central Queensland University, Rockhampton, Queensland, Australia; 2 School of Medicine & Public Health; Priority Research Centre for Physical Activity and Nutrition, Faculty of Health and Medicine, The University of Newcastle, Callaghan, New South Wales, Australia; University Sains Malaysia, MALAYSIA

## Abstract

Lifestyle behaviours significantly contribute to high levels of chronic disease in older adults. The aims of the study were to compare the prevalence and the prevalence trends of health behaviours (physical activity, fruit and vegetable consumption, fast food consumption, TV viewing, smoking and alcohol consumption), BMI and a summary health behaviour indicator score in older (65+ years) versus younger adults (18–65 years). The self-report outcomes were assessed through the Queensland Social Survey annually between 2007–2014 (*n* = 12,552). Regression analyses were conducted to compare the proportion of older versus younger adults engaging in health behaviours and of healthy weight in all years combined and examine trends in the proportion of younger and older adults engaging in health behaviours and of healthy weight over time. Older adults were more likely to meet recommended intakes of fruit and vegetable (OR = 1.43, 95%CI = 1.23–1.67), not consume fast food (OR = 2.54, 95%CI = 2.25–2.86) and be non-smokers (OR = 3.02, 95%CI = 2.53–3.60) in comparison to younger adults. Conversely, older adults were less likely to meet the physical activity recommendations (OR = 0.86, 95%CI = 0.78–0.95) and watch less than 14 hours of TV per week (OR = 0.65, 95%CI = 0.58–0.74). Overall, older adults were more likely to report engaging in 3, or at least 4 out of 5 healthy behaviours. The proportion of both older and younger adults meeting the physical activity recommendations (OR = 0.97, 95%CI = 0.95–0.98 and OR = 0.94, 95%CI = 0.91–0.97 respectively), watching less than 14 hours of TV per week (OR = 0.96, 95%CI = 0.94–0.99 and OR = 0.94, 95%CI = 0.90–0.99 respectively) and who were a healthy weight (OR = 0.95, 95%CI = 0.92–0.99 and OR = 0.96, 95%CI = 0.94–0.98 respectively) decreased over time. The proportion of older adults meeting the fruit and vegetable recommendations (OR = 0.90, 95%CI = 0.84–0.96) and not consuming fast food (OR = 0.94, 95%CI = 0.88–0.99) decreased over time. Although older adults meet more health behaviours than younger adults, the decreasing prevalence of healthy nutrition behaviours in this age group needs to be addressed.

## Introduction

Australia’s ageing population is placing a rising burden on the health care system [1]. The number of people aged 65+ years is projected to more than double from 3.2 million people in 2012 to 6.8 million by 2040 [2] and the prevalence of chronic diseases such as cardiovascular disease, type 2 diabetes, cancer and osteoporosis is 10 times greater in older Australians (65+) compared to younger (18–64) Australians [3]. Health-related behaviours such as physical activity, fruit and vegetable intake, fast food consumption, alcohol consumption, smoking and TV viewing contribute to the risk of chronic diseases and mortality [4–8]. For example, it is estimated that older adults who meet the physical activity recommendations of 150 minutes per week have a 41% lower risk of mortality over a 10 year period [9] and smoking cessation lengthens life expectancy by 4 years in adults over 55 years of age [8]. People who are overweight or obese also have a greater likelihood of chronic disease and mortality compared to someone of normal weight [10]. It is estimated that adults over 60 years of age that are obese (BMI 30–34) have an 11% greater risk of mortality [11].

Despite the association of physical activity, fruit and vegetable intake, fast food consumption, alcohol consumption, smoking and TV viewing with disease risk, a high percentage of older Australians fail to meet the health recommendations for these health behaviours. In 2014 only 45% of older adults were meeting the physical activity recommendations compared to 55% of younger adults [12], and older adults spend on average 2.3 hours per day watching TV compared to 1.7 hours in younger adults [13]. Further 72% of older adults are overweight or obese compared to 60% of younger adults [12]. Conversely a higher percentage of older adults meet the fruit and vegetable recommendations (10%) compared with 18–34 year olds (4%) [14]. The percentage of older adults who are smokers and consume more than 2 standard drinks per day (16% and 6% respectively) is typically less than the overall average (22% and 7% respectively) [14].

In the overall adult population there is evidence that physical activity, smoking and alcohol consumption behaviours are improving [12, 15, 16] whilst healthy nutrition (fruit and vegetable intake and fast food consumption) and sitting behaviours are deteriorating [17–19]. International data shows that physical activity in older adults has increased over the last 15 years [20, 21] and the proportion of older Australians meeting the recommendations rose from 17% in 2005–06, to 27% in 2011–12 [22]. The Australian Health Survey revealed that the percentage of overweight and obese adults has been steadily increasing from 50% in 2001 to 63% in 2014–15 [12, 14]. However there is limited data on trends in prevalence of healthy smoking, alcohol, nutrition and sitting behaviours in older adults. Past research has established differences in health behaviour trends by gender [15] and socioeconomic status [23]. However few studies have investigated age differences in health behaviour trends. Further, there is limited evidence of trends in total number of health behaviours met and how this differs by age. Investigations of trends in a total health behaviour indicator score is needed as the more health behaviours an individual engages in, the lower their risk of chronic disease [24].

Given the higher rates of chronic disease in older adults, their lower engagement in certain health behaviours and the ageing population it is important to examine health behaviour trends in older adults. Therefore, the aims of the current study were to 1) compare the prevalence of health behaviours and BMI in older adults (65+ years) in comparison to younger adults (18–64 years) and 2) to examine older adults’ prevalence trends from 2007–2014 for healthy physical activity, TV viewing time, nutrition (fruit and vegetable intake and fast food consumption), smoking, alcohol consumption behaviours, BMI and a total health behaviour indicator score separately to younger adults.

## Methods

### Participants and procedure

The current study pooled data from a series of separate cross-sectional surveys of adults living in Queensland, Australia. The surveys were conducted annually from 2007–2014 by the Population Research Laboratory, at CQUniversity. The Population Research Laboratory calls randomly selected Queensland household landlines each year and collects data on demographics and health behaviours via computer-assisted telephone interview (CATI). Two surveys were run in 2012 and 2013, both of which are included in the current study. Not all outcome variables were collected in every survey year. BMI, smoking and physical activity were collected from 2007–2014, alcohol consumption was collected from 2008–2014, nutrition (fruit and vegetable intake and fast food consumption) was collected from 2009–2014 and TV viewing was collected from 2007–2012. Detailed descriptions of the Queensland social survey can be found elsewhere [25]. All respondents were aged over 18 years of age, could speak and understand the English language and the sample each year was stratified by gender to match the characteristics of the Australian population. The overall sample size was 12,552, and the sample size for each individual year can be found in Table 1. Each survey was approved by CQUniversity’s Human Research Ethics Committee. Oral consent was received from participants which is common in telephone surveys and approved by the ethics committee. Annual response rates for each year were 34.7% (2007), 37.1% (2008), 41.5% (2009), 35.2% (2010), 31.9% (2011), 37.8% (2012), 39.8% (2013) and 35.9% (2014). The average was 37.1% which is comparable to those reported in other recently conducted CATI based surveys [26].

**Table 1:** Sample demographics by year.

	2007 n(%) N = 1212	2008 n(%) N = 1243	2009 n(%)N = 1292	2010 n(%) N = 1261	2011 n(%) N = 1265	2012 n(%) N = 2519	2013 n(%) N = 2537	2014 n(%) N = 1223	Total N = 12,552
**Gender**									
Male	607 (50.1)	623 (50.1)	648 (50.2)	635 (50.4)	633 (50.0)	1260 (50.0)	1288 (50.8)	611 (50.0)	6305 (50.2)
Female	605 (49.9)	620 (49.9)	644 (49.8)	626 (49.6)	632 (50.0)	1259 (50.0)	1249 (49.2)	612 (50.0)	6247 (49.8)
**Age**									
18–64	953 (79.1)	969 (78.5)	949 (73.9)	958 (76.6)	924 (73.6)	1744 (69.6)	1740 (69.1)	807 (66.3)	9044 (72.5)
65+	252 (20.9)	266 (21.5)	336 (26.1)	293 (23.4)	331 (26.4)	760 (30.4)	777 (30.9)	411 (33.7)	3426 (27.5)
**Employment**									
Yes	700 (58.2)	722 (58.2)	713 (55.4)	744 (59.0)	731 (57.9)	1418 (56.5)	1435 (56.7)	675 (55.2)	7138 (57.0)
No	502 (41.8)	519 (41.8)	574 (44.6)	517 (41.0)	532 (42.1)	1093 (43.5)	1098 (43.3)	547 (44.8)	5382 (43.0)
**Education**									
0–12 years	614 (52.1)	642 (52.0)	669 (52.3)	613 (49.0)	592 (47.3)	1159 (46.5)	1126 (44.7)	550 (45.4)	5965 (48.1)
13+ years	565 (47.9)	592 (48.0)	611 (47.7)	637 (51.0)	659 (52.7)	1333 (53.5)	1391 (55.3)	661 (54.6)	6449 (51.9)
**Location**									
City	597 (49.6)	627 (50.6)	635 (49.2)	656 (52.2)	771 (61.1)	1487 (59.2)	1586 (62.7)	730 (59.9)	7089 (56.6)
Town	319 (26.5)	328 (26.5)	349 (27.1)	321 (25.5)	276 (21.9)	617 (24.6)	572 (22.6)	257 (21.1)	3039 (24.3)
Rural	287 (23.9)	285 (23.0)	306 (23.7)	280 (22.3)	215 (17.0)	408 (16.2)	373 (14.7)	232 (19.0)	2386 (19.1)

### Measures

*Sociodemographic factors* included gender, age (younger, 18–64 years and older, 65+ years), education (13 years or less or over 13 years) and employment (employed or not employed). The ‘not employed’ category includes respondents who were retired and those who engaged in full time home duties. Participants’ location was also collected by asking participants ‘do you presently live in a city, town, or rural area?’ In Australia, a city is used to describe a large metropolitan city (e.g. Sydney or Brisbane), a town is used to describe small cities, usually under 200,000 people (e.g. Rockhampton, Mackay) and a rural area is used to describe a remote area (e.g. farming regions). These location categories have been used in past research [27].

*Body Mass Index (BMI)* was calculated by dividing participants’ self-reported weight by height squared (kg/m^2^). Following the Australian classifications for overweight and obesity [28] participants were dichotomised based on their BMI (normal < 25; and overweight or obese ≥ 25).

*Physical activity* was measured using the Active Australia Questionnaire which assess the duration and frequency of recreational and transport walking, moderate and vigorous intensity physical activity [29]. Total physical activity was calculated by summing the time spent in walking, moderate activity and vigorous activity (weighted by two) according to specified scoring guidelines [29]. The Active Australia Questionnaire has demonstrated acceptable reliability (ICC = 0.64) [29] and criterion validity (r = 0.61) [30]. In line with the Australian physical activity recommendations, which recommend that adults engage in at least 150 minutes of physical activity per week over at least 5 sessions [31], participants were classified as inactive (<150 minutes and/or < 5 sessions) or active (≥150 minutes and ≥5 sessions).

*Fruit and vegetable intake* was assessed using two items. First portion sizes was explained to participants. They were then asked ‘How many serves of vegetables do you eat on a usual day?’ and ‘How many serves of fruit do you eat on a usual day?’ A single binary outcome was created based on whether or not participants were meeting the Australian recommendations of ≥5 servings of vegetables and ≥2 fruit [32].

*Fast food consumption* was assessed using a single item: ‘In the last week (the last 7 days), how many times did you eat something from a fast-food restaurant like McDonald’s, Hungry Jacks, KFC, etc? This also includes other fast-food and takeaway such as fish and chips, Chinese food and pizza for example’. The only recommendations the Australian government provides on fast food consumption is to limit intake [32]. As slightly over half the sample did not consume any takeaway in the week prior to the survey, fast food consumption was dichotomised into those who did, and those who did not consume any takeaway.

*Smoking status* was assessed using a single item: ‘Are you presently a smoker?’ which had a binary response option of yes or no.

*Alcohol intake* was assessed by asking participants three questions: ‘during the past 30 days, have you had at least one drink of any alcoholic beverage?’ and if yes ‘during the past 30 days, how many days did you have at least one drink of any alcoholic beverage?’ and during the past 30 days, on the days when you drank, about how many drinks did you drink on average?’ Alcohol consumption was dichotomised based on whether participants were meeting the Australian recommendations [33] of no more than two alcoholic drinks per day.

*TV viewing time* was assessed by asking respondents ‘In hours and/or minutes, what do you estimate was the total time that you spent sitting and watching television in the last week?’ TV viewing time was dichotomised into under 14 and over 14 hours per week due to the association of more than 2 hours of TV viewing time per day with an increased risk of cardiovascular disease and mortality [5, 34].

A *total health behaviour* indicator variable was calculated by summing the number of health behaviours participants met. The survey years 2007 and 2008 were not included in the total health behaviour analysis as data on alcohol consumption, fruit and vegetable intake and fast food consumption were not collected in these years. TV viewing time was not included as it was not collected in the 2013 and 2014 surveys. Therefore the total health behaviour indicator was based on the number individuals who were engaging in healthy physical activity, nutrition (fruit and vegetable intake and fast food consumption), smoking and alcohol consumption behaviours in each survey year from 2009 to 2014. A low percentage of participants met 0 or 1 health behaviour (4.4%) or 5 behaviours (5%), thus the total health behaviour scores were collapsed into 3 categories; 1) meeting 0–2 health behaviours, 2) meeting 3 health behaviours and 4) meeting 4–5 health behaviours.

### Data analysis

**Fig 1:**
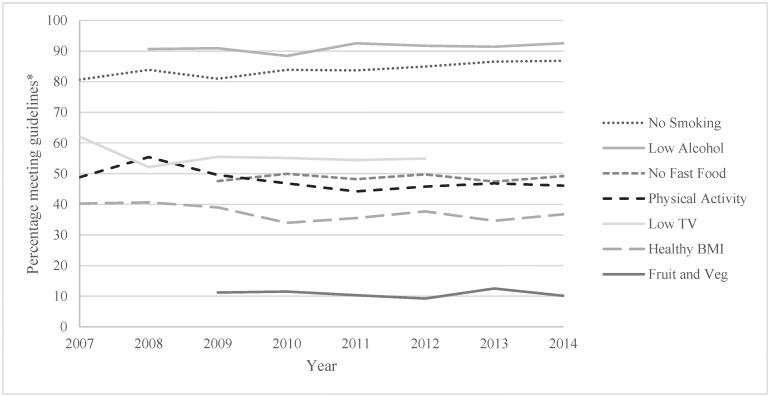
Adjusted trend lines of health behaviours and BMI in younger (18–64 years) Queensland adults. *adjusted for gender, education, employment and location.

**Fig 2:**
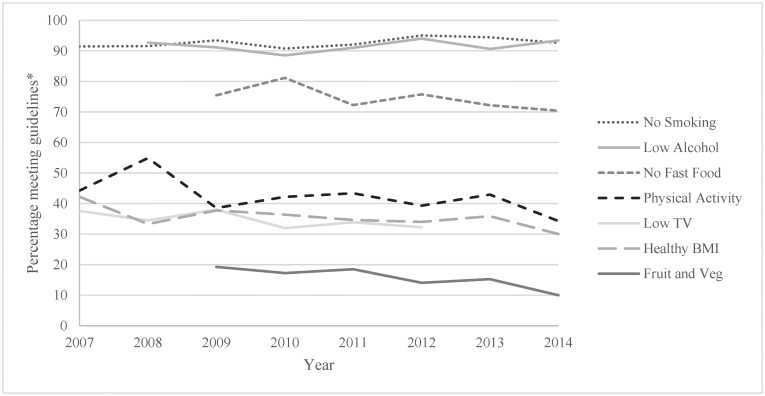
Adjusted trend lines of health behaviours and BMI in older (65+ years) Queensland adults. *adjusted for gender, education, employment and location.

Descriptive statistics were calculated for demographics and health behaviours by each year and in total (Tables 1 and 2). First, 7 logistic regression analyses with age group (18–64 and 65+) as the independent variable and each of the 6 health behaviours and BMI as the dependent variables were conducted. A multinomial regression was also conducted for the total health behaviour indicator scores with age group as the independent variable. These analyses were conducted to compare the prevalence of each health behaviour, BMI and total health behaviour indicator scores in older versus younger adults in all years combined (Aim 1). Second, 7 logistic regression analyses were conducted with year (continuous) as the independent variable and BMI and each of the 6 health behaviours as the dependent variables. This was done for both older and younger adults separately. A multinomial regression was also conducted with year as the independent variable and health behaviour indicator scores as the dependant variable. This was also done for both older and younger adults separately. These analyses were conducted to examine the trends in BMI, each health behaviour and total health behaviour indicator scores in younger adults and older adults separately (Aim 2). For all analyses the younger age group was used as the reference category for the independent variable age and the unhealthy or high risk behaviours were used as the reference category for the health behaviour outcome variables. The trend analyses for nutrition (fruit and vegetable consumption and fast food intake) and TV viewing was limited to 6 years, and alcohol consumption to 7 years due to these behaviours not being collected every survey year. A series of chi square analyses were conducted to test for significant group differences between demographics for each individual health behaviour, BMI as well as total health behaviour indicator scores. Any demographic factors found to be significantly (p <.05) different across a health behaviour, BMI or total health behaviour indicator scores were entered as a covariate into the regression model for that outcome variable. This strategy ensures that the covariates are accounted for appropriately without over-inflating the models [35]. The covariates entered into each regression analysis are listed in Table 3. The prevalence trends for healthy behaviours and BMI presented in Figs 1 and 2 were adjusted by weighting the prevalence of each year for gender, education, employment and location of that year’s survey. All analyses were conducted using IBM SPSS version 23.

**Table 2:** Percentage of Queensland adults engaging in healthy behaviours and BMI by year.

	2007 n(%) N = 1212	2008 n(%) N = 1243	2009 n(%) N = 1292	2010 n(%) N = 1261	2011 n(%) N = 1265	2012 n(%) N = 2519	2013 n(%) N = 2537	2014 n(%) N = 1223	Total N = 12,552
**Meeting physical activity recommendations**									
Total	571 (47.9)	671 (55.6)	598 (46.4)	584 (46.6)	567 (45.0)	1121 (44.6)	1173 (46.5)	529 (43.3)	5814 (46.7)
Younger adults	455 (48.5)	521 (55.5)	469 (49.5)	455 (47.6)	414 (45.0)	808 (46.4)	823 (47.5)	377 (46.7)	4322 (48.1)
Older adults	112 (45.2)	147 (56.5)	127 (37.8)	125 (43.1)	148 (44.8)	305 (40.3)	337 (43.7)	148 (36.1)	1449 (42.6)
**Meeting fruit and vegetable recommendations**									
Total			171 (13.3)	168 (13.4)	159 (12.6)	276 (11.0)	347 (13.7)	130 (10.6)	1251 (12.4)
Younger adults			106 (11.2)	114 (11.9)	98 (10.6)	163 (9.4)	221 (12.7)	87 (10.8)	789 (11.1)
Older adults			64 (19.1)	52 (17.8)	60 (18.2)	112 (14.7)	123 (15.9)	43 (10.5)	454 (15.6)
**Did not consume fast food in the prior week**									
Total			723 (56.0)	727 (57.7)	696 (55.0)	1455 (57.8)	1423 (56.1)	689 (56.4)	5713 (56.6)
Younger adults			463 (48.8)	485 (50.6)	453 (49.0)	864 (49.6)	842 (48.4)	399 (49.5)	3506 (49.2)
Older adults			254 (75.6)	235 (80.2)	238 (71.9)	581 (76.4)	568 (73.1)	288 (70.2)	2164 (74.4)
**Non- Smoker**									
Total	1006 (83.1)	1068 (86.0)	1089 (84.4)	1076 (85.4)	1092 (86.4)	2224 (88.3)	2263 (89.3)	1087 (89.0)	10905 (87.0)
Younger adults	771 (81.0)	818 (84.5)	768 (81.1)	801 (83.7)	781 (84.5)	1487 (85.3)	1515 (87.1)	701 (87.0)	7642 (84.6)
Older adults	228 (90.8)	243 (91.4)	315 (93.8)	265 (90.4)	301 (91.2)	723 (95.1)	729 (93.9)	382 (92.9)	3186 (93.1)
**Meeting alcohol recommendations**									
Total		1124 (91.2)	1166 (90.8)	1116 (88.7)	1158 (92.2)	2321 (92.5)	2307 (91.3)	1130 (92.9)	10322 (91.5)
Younger adults		870 (90.7)	854 (90.6)	850 (89.0)	851 (92.8)	1598 (91.8)	1585 (91.5)	744 (92.8)	7352 (91.3)
Older adults		247 (93.2)	305 (91.3)	258 (88.1)	297 (90.3)	708 (93.8)	702 (90.7)	381 (93.2)	2898 (91.7)
**Under 14 hours of TV**									
Total	689 (57.0)	603 (48.7)	662 (51.4)	633 (50.3)	621 (49.2)	1209 (48.1)			4350 (49.6)
Younger adults	591 (62.2)	508 (52.6)	530 (56.0)	536 (56.0)	503 (54.7)	957 (55.0)			3625 (56.0)
Older adults	94 (37.5)	91 (34.2)	130 (38.9)	93 (31.8)	112 (33.8)	247 (32.6)			767 (34.4)
**Healthy BMI**									
Total	471 (42.1)	449 (39.3)	466 (38.8)	408 (35.3)	419 (35.9)	867 (36.9)	841 (35.5)	400 (35.4)	4321 (37.1)
Younger adults	369 (41.8)	361 (40.5)	346 (39.0)	309 (34.7)	303 (35.5)	625 (38.2)	576 (35.1)	282 (37.8)	3171 (37.6)
Older adults	99 (42.5)	86 (35.2)	119 (38.8)	97 (37.0)	112 (36.4)	240 (33.8)	256 (35.7)	118 (31.2)	1127 (35.7)
**Total Health Behaviours**									
Total 3 health behaviours			492 (38.6)	470 (37.7)	476 (38.1)	1027 (41.1)	1021 (40.7)	468 (38.6)	3954 (39.6)
Total 4–5 health behaviours			423 (33.2)	423 (33.9)	410 (32.8)	809 (32.4)	845 (33.7)	403 (33.3)	3313 (33.2)
Younger 3 health behaviours			347 (37.1)	363 (38.3)	358 (39.3)	691 (39.9)	700 (40.6)	312 (39.0)	2771 (39.3)
Younger 4–5 health behaviours			287 (30.7)	288 (30.3)	262 (28.7)	500 (28.9)	522 (30.3)	246 (30.8)	2105 (29.8)
Older 3 health behaviours			141 (42.3)	102 (35.2)	116 (35.5)	325 (43.2)	312 (40.7)	153 (37.6)	1149 (40.0)
Older 4–5 health behaviours			134 (40.2)	132 (45.5)	143 (43.7)	305 (40.6)	314 (40.9)	156 (38.3)	1184 (41.2)

**Table 3:** Odds ratios of health behaviours and BMI by year and age group.

	Prevalence all years		Trends			
	Age group comparisonReference: younger adultsOR (95% CI)	P value	Trends in younger adults OR (95% CI)	P value	Trends in older adultsOR (95% CI)	P value
**Physical activity**^a^						
Meeting recommendations	0.86 (0.78–0.95)	.004	0.97 (0.95–0.98)	<.0001	0.94 (0.91–0.97)	<.0001
Not meeting recommendations	Reference		Reference		Reference	
**Fruit and vegetable**^b^						
Meeting recommendations	1.43 (1.23–1.67)	<.0001	1.01 (0.96–1.06)	.75	0.90 (0.84–0.96)	.001
Not meeting recommendations	Reference		Reference		Reference	
**Fast food**^c^						
No	2.54 (2.25–2.86)	<.0001	1.00 (0.97–1.03)	.97	0.94 (0.88–0.99)	.03
Yes	Reference		Reference		Reference	
**Smoking status**^d^						
No	3.02 (2.53–3.60)	<.0001	1.05 (1.02–1.07)	.001	1.04 (0.98–1.12)	.19
Yes	Reference		Reference		Reference	
**Alcohol**^e^						
Meeting recommendations	1.08 (0.93–1.26)	.32	1.05 (1.01–1.10)	.02	1.02 (0.95–1.09)	.63
Not meeting recommendations	Reference		Reference		Reference	
**TV time**^f^						
Under 14 hours	0.65 (0.58–0.74)	<.0001	0.96 (0.94–0.99)	.01	0.94 (0.90–0.99)	.03
Over 14 hours	Reference		Reference		Reference	
BMI^g^						
Healthy	1.11 (1.00–1.24)	.05	0.96 (0.94–0.98)	<.0001	0.95 (0.92–0.99)	.005
Overweight or obese	Reference		Reference		Reference	
**Total Health Behaviours**^h^						
Meeting 4–5 health behaviours	2.27 (1.95–2.64)	<.0001	1.00 (0.96–1.04)	.97	0.96 (0.90–1.03)	.24
Meeting 3 health behaviours	1.73 (1.51–1.99)	<.0001	1.02 (0.99–1.06)	.22	0.98 (.92–1.05)	.52
Meeting 0–2 health behaviours	Reference		Reference		Reference	

^a^. n = 11364, years 2007–2014. Controlled for BMI, gender, location, employment and education.

^b^. n = 9995, years 2009–2014. Controlled for gender and employment.

^c^. n = 9237, years 2009–2014. Controlled for BMI, gender, location, employment and education.

^d^. n = 11428, years 2007–2014. Controlled for BMI, gender, location, employment and education.

^e^. n = 10427, years 2008–2014. Controlled for BMI and gender.

^f^. n = 7996, years 2007–2012. Controlled for BMI, gender, location and employment.

^g^. n = 11467, years 2007–2014. Controlled for gender, employment and education.

^h^. n = 9155, years 2009–2014. Controlled for BMI, gender, location, employment and education.

## Results

Approximately, half of the sample were males (50%), and 28% were 65 years or older. Just over half of the sample was employed in paid work (57%), had more than 13 years of education (52%) and lived in a city (57%) (Table 1).

Approximately half of the sample were meeting the physical activity recommendations (47%), had not consumed fast food in the week prior (57%) and were engaging in less than 14 hours of TV viewing per week (50%). Over half were and were overweight or obese (63%) and a high percentage were non-smokers (87%) and were meeting the alcohol recommendations (91%). A low percentage were meeting the fruit and vegetable recommendations (12%) (Table 2).

Table 3 presents the odds ratios of the first model comparing older adults’ health behaviours, BMI and total health behaviour indicator scores to younger adults across all years, and the second model shows the trends in health behaviours, BMI and total health behaviour indicator scores stratified by age. Figs 1 and 2 presents the adjusted proportion of younger (Fig 1) and older (Fig 2) adults engaging in health behaviours and of healthy weight each year. The proportion of older adults engaging in healthy fruit and vegetable intake was approximately 1.4 times higher than younger adults, the proportion of low fast food consumers was around 2.5 times higher in older adults and the proportion of non-smokers was around 3 times higher in older adults. However older adults were 14% less likely to meet the physical activity recommendations and were 35% less likely to have a low screen time. The proportion of older adults meeting 3 health behaviours was 1.7 times higher and the proportion meeting at least 4 health behaviours was 2.3 times higher than younger adults.

The odds of meeting the physical activity recommendations significantly dropped by 6% per year in older adults and 3% per year in younger adults between 2007 and 2014. The odds of engaging in less than 14 hours of TV viewing per week was estimated to drop by 6% per year in older adults and 4% per year in younger adults between 2007 and 2012 which were statistically significant. The odds of being a healthy weight significantly reduced by 5% per year in older adults and 4% per year in younger adults between 2007 and 2014. The remaining trends varied by age group. In the younger adult age group the odds of not smoking significantly increased by 5% per year between 2007–2014 and the odds of meeting the alcohol recommendations significantly increased by 5% per year between 2008–2014. In older adults the odds of meeting the fruit and vegetable recommendations significantly decreased by 10% per year between 2009–2014 and the odds of not eating fast food in the week prior significantly decreased by 6% per year between 2009–2014.

## Discussion

The aims of the study were to investigate the health behaviours of older adults in Queensland and their prevalence trends between 2007 and 2014. Older adults are performing better on many health behaviours in all years combined, however it is concerning that healthy nutrition (fruit and vegetable intake and fast food consumption) behaviours decreased in older adults over the survey years. It is also concerning that the percentage meeting the physical activity recommendations, engaging in less than 14 hours of TV viewing time per week and of healthy weight decreased in both age groups. Whilst there were higher rates of non-smokers in the older adult age group, the proportion of non-smokers and low alcohol consumers in the younger age group increased over the survey years. The proportion of Queensland adults meeting health behaviour recommendations observed in the current study are mostly in line with the latest Australian Health Survey findings [12], including the high percentage of inactive people and people who are overweight or obese (53% and 63% respectively).[12] However the percentage of people meeting the fruit and vegetable (12%) and alcohol recommendations (91%) differed to the Australian Health Survey’s results of the percentage of people meeting fruit and vegetable (5%) and alcohol recommendations (83%) [12]. These differences may be due to the older age of the sample in the Queensland Social Survey. Past research has shown older adults are more likely to meet the fruit and vegetable and alcohol guidelines [14]. The differences may also be due to differences in data collection methods. The Australian health survey used a 24 hour food intake recall conducted via interview where participants were asked to recall everything they had consumed in the previous 24 hours. Participants were prompted to provide more detail if needed and fruit and vegetable servings were then calculated [22]. For the current study participants were asked to recall the number of fruit and vegetable servings they consume on a usual day. Although the size of a serving was explained there may be a higher risk of recall or social desirability bias using this method leading to inflated estimates of fruit and vegetable intake. [22]The high percentages of Queensland adults not meeting health recommendations particularly for physical activity, nutrition (fruit and vegetable intake and fast food consumption),TV viewing and BMI confirms that these are a significant public health issues which needs to be addressed in order to reduce chronic disease levels [4, 36].

In line with past research, the current findings indicate that a higher percentage of older adults compared to younger adults are engaging in healthy nutrition (fruit and vegetable intake and fast food consumption) behaviours [14], are non-smokers [14], are meeting 3 health behaviours and are meeting at least 4 out of the 5 health behaviours. Further, in line with past research [37] older adults were more likely to be of healthy weight compared to younger adults which was approaching significance. This may be a result of healthy habits older adults established when they were younger [17]. It could also be due to older adults becoming more aware of their health behaviours as they are at an age where they are at a higher risk of developing chronic diseases resulting from health behaviours [38]. Research has shown that chronic disease diagnosis in older adults is a key ‘teachable moment’ where they are more likely to improve their health behaviours, in particular smoking [38]. This may be the reason for the reduced smoking in older adults, as smoking prevalence used to be higher when they were still in the younger age group [16]. Alternatively it could be due to a survival effect where the high mortality in smokers leads to few remaining older adults who smoke [8]. The lower alcohol consumption observed in older adults in past research was not seen in the current sample. Older adults were however significantly less likely to meet the physical activity recommendations and engage in less than 14 hours of TV viewing putting them at increased risk of cardiovascular disease and mortality. This could be due to older adult’s loss of mobility and fewer work commitments [34, 39]. Despite older adults demonstrating healthier nutrition and smoking behaviours and BMI, there is a need to address their low physical activity and high TV viewing.

The trends of many health behaviours differed between younger and older adults. No trend in the proportion meeting fruit and vegetable recommendations was observed in younger adults, however the proportion of older adults meeting the fruit and vegetable recommendations and not consuming fast food decreased in line with past research [12, 17]. Although a higher percentage of older adults met the fruit and vegetable recommendations overall, the percentage dropped between 2009–2014 and in 2014 was equal to the low percentage of younger adults meeting the fruit and vegetable recommendations (11%). This finding may be due to older adults having increasing barriers to healthy nutrition. An Australian study found that fast food consumption was associated with running out of money to buy groceries and difficulties lifting groceries [40]. These risk factors are particularly relevant to older adults who are more likely to have lower incomes [41] and are more likely to have physical restrictions [42]. These risk factors might be increasing due to rising fresh fruit and vegetable costs [43], and increased functional limitations in older adults due to Australians increasing lifespans [42]. Further, past research has demonstrated that older adults improve their health behaviours when they retire as they have more time to look after their health [44], but as the age of retirement is increasing it may be contributing to the decline in the percentage of older adults engaging in healthy nutrition behaviours [45]. These trends may become a significant issue for the health of older adults if they continue.

Whilst the trend of a decreasing number of both younger and older adults with low TV viewing time and of normal weight is in line with past research [19], the trend of a decreasing number of both younger and older adults meeting the physical activity recommendations is not in line with past research which found an increasing trend in many high income countries [15, 20, 21]. This difference may be due to when previous studies were conducted or due to the current study being conducted in Queensland. For example, many of the large trend studies were conducted several years ago (e.g., 10 years ago) [20]. The findings from this study may have detected a change in the original trends, which may have switched from increasing to decreasing proportions of the population meeting recommendations. Further the previous research was conducted in other locations [21], therefore the decreasing trend may only be occurring in Queensland adults. [20, 21]The trend in both younger and older adults of decreasing physical activity and increasing TV viewing time is a concerning finding and may be contributing to the decreasing trend of healthy weight in younger and older adults. This demonstrates the need for continuous monitoring and targeted health promotion initiatives for physical activity and sedentary behaviour in Queensland adults. Despite the decreasing trend for physical activity, it varied across the years. In particular there was an increase in both younger and older adults meeting the recommendations in 2008. The reason for this variation in activity levels is unknown. The trend of decreased alcohol consumption [12] and smoking [16] seen in past research was observed in younger but not older adults. It is possible that the decreasing alcohol consumption and smoking seen in past research is predominately due to having younger participants in previous studies. The decreasing rates of smoking in younger adults is likely to be due to the increased legislation to target smoking such as increased taxation, restricted smoking areas and plain packaging [16]. Further, there was a steep increase in the percentage of younger adults meeting the alcohol recommendations in 2011 which continued to 2014. This may be due to the liquor amendment bill implemented in 2010 which included stricter alcohol licencing laws to reduce availability of alcohol [46]. Overall it is positive that smoking and alcohol behaviours are improving in younger adults, particularly due to the higher rates of smoking in this age group.

This study provides evidence for the health behaviour and BMI trends in younger and older Queensland adults which is beneficial for health promotion initiatives. A major strength of the study was the large number of participants (n = 12,552), however only 28% of the sample were over 65 years of age (n = 3,426) which may reduce the precision of the prevalence estimates in this age group. Further, not all of the health behaviours were included in every year so only 6 or 7 year trends could be identified for nutrition, alcohol and TV viewing. The surveys were cross sectional so changes in health behaviours within individuals could not be measured. The data collected was also self-reported which is likely to have a higher error than objective measures and may have resulted in social desirability bias. Further, only an overall measure of servings of fruits and vegetables was used so a detailed analysis of types of fruits and vegetables consumed could not be conducted. As the survey was conducted in Queensland adults the findings cannot be generalised to other states or countries. Trends in health behaviours of older and younger adults in other locations needs to be investigated. Additional studies with objective measures of health behaviours and a larger sample of adults over 65 years of age is required.

Overall the findings revealed that on average over all survey years, a higher percentage of older adults were meeting the fruit and vegetable recommendations, were not eating fast food, were non-smokers, were meeting 3 out of 5 health behaviours and were meeting at least 4 out of 5 health behaviours compared to younger adults. However, a higher percentage of older adults were inactive and had a high TV viewing time. Further, the age gap in nutrition behaviours is closing as an increasing trend of fast food consumption and a decreasing trend of fruit and vegetable intake was seen in older adults. The percentage of both younger and older adults with healthy TV viewing and physical activity behaviours and of healthy weight was decreasing in both age groups. It is positive that older adults were engaging in more health behaviours than younger adults in all years combined. However the trend of a decreasing proportion of older adults engaging in healthy nutrition behaviours and a decreasing proportion of both younger and older adults engaging in healthy physical activity and TV viewing behaviours and of healthy weight supports the need for health promotion initiatives targeting these behaviours.
